# Aquaporins Mediate Silicon Transport in Humans

**DOI:** 10.1371/journal.pone.0136149

**Published:** 2015-08-27

**Authors:** Alexandre P. Garneau, Gabriel A. Carpentier, Andrée-Anne Marcoux, Rachelle Frenette-Cotton, Charles F. Simard, Wilfried Rémus-Borel, Luc Caron, Mariève Jacob-Wagner, Micheline Noël, Jonathan J. Powell, Richard Bélanger, François Côté, Paul Isenring

**Affiliations:** 1 L’Hôtel-Dieu de Québec Research Center, Department of Medicine, Faculty of Medicine, Université Laval, Québec City, Québec, Canada; 2 Department of Phytology, Faculty of Sciences of Agriculture and Alimentation, Laval Université Laval, Québec City, Québec, Canada; 3 Medical Research Council Human Nutrition Research, Elsie Widdowson Laboratory, Cambridge, United Kingdom; University of Minho, PORTUGAL

## Abstract

In animals, silicon is an abundant and differentially distributed trace element that is believed to play important biological functions. One would thus expect silicon concentrations in body fluids to be regulated by silicon transporters at the surface of many cell types. Curiously, however, and even though they exist in plants and algae, no such transporters have been identified to date in vertebrates. Here, we show for the first time that the human aquaglyceroporins, i.e., AQP3, AQP7, AQP9 and AQP10 can act as silicon transporters in both *Xenopus laevis* oocytes and HEK-293 cells. In particular, heterologously expressed AQP7, AQP9 and AQP10 are all able to induce robust, saturable, phloretin-sensitive silicon transport activity in the range that was observed for low silicon rice 1 (lsi1), a silicon transporter in plant. Furthermore, we show that the aquaglyceroporins appear as relevant silicon permeation pathways in both mice and humans based on 1) the kinetics of substrate transport, 2) their presence in tissues where silicon is presumed to play key roles and 3) their transcriptional responses to changes in dietary silicon. Taken together, our data provide new evidence that silicon is a potentially important biological element in animals and that its body distribution is regulated. They should open up original areas of investigations aimed at deciphering the true physiological role of silicon in vertebrates.

## Introduction

**Fig 1:**
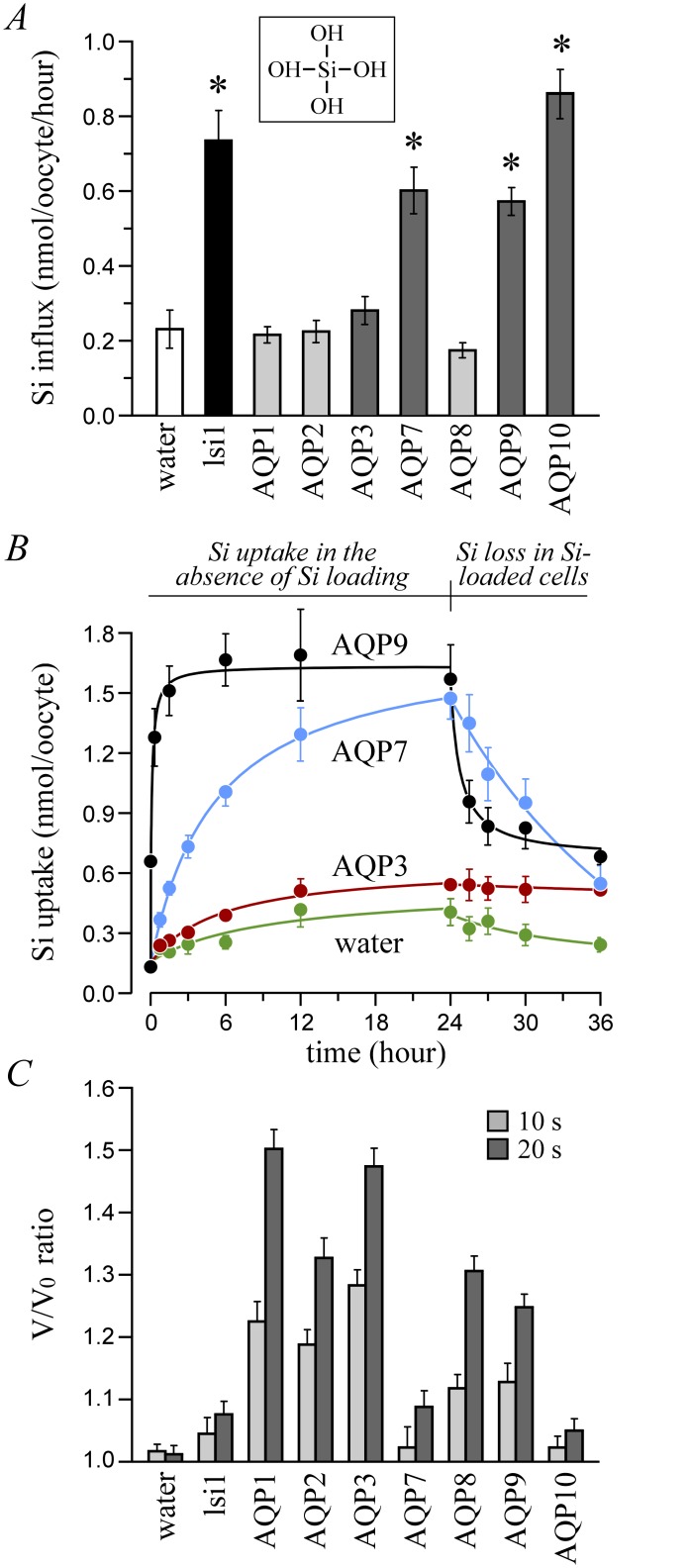
Standard Si transport studies in *Xenopus laevis* oocytes. (A) Si influx. Oocytes incubated for 30 min (AQP9), 60 min (AQP10) or 90 min (lsi1 and other AQPs) in B1 medium (see Table 2) + 2 mM H_4_SiO_4_ were assayed for Si content. Data are presented as means ± S.E. of 3 measurements among 4 experiments using * to indicate that they are significantly different compared to the controls. The image inserted on the top is to show the chemical structure of H_4_SiO_4_. (B) Si uptake and loss *vs*. time. Oocytes incubated for 0 to 36 h in B1 medium (± 2 mM H_4_SiO_4_ from 0 to 24 h and no H_4_SiO_4_ from 24 to 36 h) were assayed for Si content. Data are presented as means ± S.E. of 3 measurements among 5 or more experiments. Compared to the controls, data for AQP7 and AQP9 are all significantly different beyond the zero point, and data for AQP3 are all significantly different from 6 to 36 h. (C) Water transport. Oocytes were assayed for cell volume measurements during a 20-s incubation step in distilled water added with ~10 mM sucrose. Data are expressed as *n*-fold increases in cell volumes (V) relative to initial cell volumes (V_0_) and are presented as means ± S.E. of 5 oocytes among 3 experiments. They are all significantly different compared to the controls at 20 s.

Silicon (Si) is the richest element of the Earth's soil and crust after oxygen, and the most abundant trace element in human after iron and zinc [1,2]. Orthosilicic acid (H_4_SiO_4_) in natural water, beer and digested plants is the readily available source of Si to man. H_4_SiO_4_ is also the main Si species in human (see chemical formula in Fig 1) [3–9]. In blood, it is largely unbound except for a small pool that forms complexes with Al or Fe at circumneutral pH and its concentration is between 10 and 50 μM [3–5,9]. Outside of blood, H_4_SiO_4_ is largely bound to glycosaminoglycans and is particularly abundant in aorta, trachea, tendon, bone and skin [6–9].

Although mechanistic insight is still lacking, growing evidence suggests that Si plays key biological roles in mammals. Examples are as follows: 1) osteoblast-like cells exhibit increased differentiation, prolyl hydroxylase activity and collagen synthesis in Si-rich medium [10,11]; 2) appendicular bone growth in developing rat is accelerated under Si-poor diets [12,13]; 3) higher adjusted hip bone mineral density in men and premenopausal women is associated with higher Si intake [14]; and 4) recent studies point towards the importance of urinary conservation to maintain active body Si levels in rats [13]. Hence, Si in mammals does not appear to behave as a simple *in vivo* contaminant, implying that its tissue levels must be tightly regulated through Si transporters and Si-responsive elements [13–15].

A Si transporter termed low Si rice 1 (lsi1) was recently identified in plant for the first time by Ma *et al*. [16]. It was found to provide the root exodermis with a permeation pathway for the transfer of H_4_SiO_4_ from soil to phloem. Interestingly, the closest homolog of lsi1 in humans was also found to include a member of the water channel (aquaporin; AQP) family, although global amino acid conservation was still relatively low (less than 25% identity). Of notice, no data was reported then to indicate whether lsi1 could transport water, H_2_O_2_, nitrates, urea or arsenite as some of the known human AQPs [17].

The purpose of this study was to identify H_4_SiO_4_ transport systems in humans by relying on the premise that a number of AQPs could have retained the ancestral ability of acting as permeation pathways for Si beyond the eumatazoal divergent point. Nine human AQPs selected out of the thirteen reported to exist [17] were thus tested for their ability to promote Si movement across the lipid bilayer. Three such AQPs belonged to the conventional AQP subfamily (AQP1, AQP2 and AQP8), four to the so-called aquaglyceroporin (AQGP) subfamily (AQP3, AQP7, AQP9 and AQP10), and one to the so-called super AQP (S-AQP) subfamily (AQP11).

Our results showed that some, if not all of the AQGP subfamily members did retain the ancestral ability of inducing saturable Si transport based on kinetic determinations. We have thus uncovered for the first time Si transporters in humans, providing new evidence for the biological relevance of Si in a wider variety of species.

## Materials and Methods

### Plasmids

All of the cDNA inserts required for this work were amplified by PCR from commercially available kidney, heart, pancreas, liver or brain cDNA libraries of human origin and were cloned in the *Xenopus laevis* oocyte expression vector called Pol1 [15] or in the mammalian expression vector called pCDNA3. AQP7 in Pol1 was also tagged with the *c-Myc* epitope by inserting pairs of oligonucleotides in front of the first ATG and used as a template to generate AQP7_G264V_ from mutagenic primers. Addition of an epitope tag to the other AQPs led water transport activity to decrease substantially so that most of the studies were carried out using the untagged proteins. All of the sequences used for amplification or priming are listed in Table 1.

**Table 1:** Oligonucleotides used.

Gene	Direction	Sequence (5’ → 3’)
Cloning of AQPs		
AQP1	Forward	GAATTCATGGCCAGCGAGTTCAAG
	Reverse	AAGCTTCCTACGTGGATGCCCGGG
AQP2	Forward	CATAGAATTCGAGCATCCTGGCCCTGAG
	Reverse	CTACTTCATAAAGCTTTTCAGACCTGCGGGAGAG
AQP3	Forward	GCGTGAATTCCATGGGTCGACAGAAGGAG
	Reverse	GCGTAAGCTTGGACAGTCAGTGGATGCTCAAG
AQP7	Forward	GCCCGAATTCACATGGTTCAAGCATCCG
	Reverse	GCCGAAGCTTTATTGGGGAATGGATGGG
AQP7_G264V_	Forward	GCACCACTTCTGGTTGCCTATCTAGGTGG
	Reverse	CCACCTAGATAGGCAACCAGAAGTGGTGC
AQP8	Forward	GCCCGAATTCTCCGATGTTTGTGCCATCTGATCC
	Reverse	GCGTAAGCTTCCACGAGCTCTGCTTCACCG
AQP9	Forward	GCCCGAATTCCAGAGAAGCCCCAAGATGCAGCC
	Reverse	GCCGTCTAGACCCAAACTGACTGCAAATCCAGAGC
AQP10	Forward	GTAGAATTCCATGGTCTTCACTCAGGCCCCG
	Reverse	CATCTAGAAATCATAGCTTACACTCCAGCATCTGAGC
AQP11	Forward	CTAAGAATTCAGACCGCCTCCTACCCAGAG
	Reverse	CATATCTAGACCGGTGTTTTCCATATGAGG
Quantitative Real-Time PCR		
AQP1	Forward	TACATCATCGCCCAGTGTGT
	Reverse	TGCAGAGTGCCAATGATCTC
AQP3	Forward	AAGCTGCCCATCTATGCACT
	Reverse	CAACGATGGCCAGTACACAC
AQP7	Forward	CTTCAGGTCCACCCACAACT
	Reverse	CCAAAACCCAAGTTGACACC
AQP9	Forward	TTGGAAGGATGGAGTGGTTC
	Reverse	GGCACGGATACAAATGGTTT
GAPDH	Forward	ACCCAGAAGACTGTGGATGG
	Reverse	CACATTGGGGGTAGGAACAC

### Transport and expression studies

Several of the AQP family members were tested for their ability to act as H_4_SiO_4_ transporters in both *Xenopus laevis* oocytes and HEK-293 cells. Experimental procedures are described further in the Fig legends and the media used in Table 2.

**Table 2:** Composition of media.

	Na^+^	K^+^	Cl^−^	Ca^2+^	Mg^2+^	SO_4_^2−^	NO_3_^−^	Bic	Si or Ge^a^	HEP	NMG	Ace	Glu	Suc	pH	Osm
**B1**	96	1	85	0.75	0.8	0.8	0.65	2.5	0–2	10	0	0	0	0	7.4	200
**B2a**	74	1	62	0.55	0.6	0.6	0.5	2	2	7.5	0	0	0	0	7.4	150
**B2b**	74	1	62	0.55	0.6	0.6	0.5	2	2	7.5	0	0	0	50	7.4	200
**B2c**	74	1	62	0.55	0.6	0.6	0.5	2	2	7.5	0	0	0	100	7.4	250
**B3a**	6	1	85	0.75	0.8	0.8	0.65	2.5	2	10	90	0	0	0	7.4	200
**B3b**	96	0	85	0.75	0.8	0.8	0.65	2.5	2	10	1	0	0	0	7.4	200
**B3c**	96	1	5	0.75	0.8	0.8	0.65	2.5	2	10	0	0	80	0	7.4	200
**B3d**	96	1	5	0.75	0.8	0.8	0.65	2.5	2	10	0	0	80	0	^b^	200
**B3e**	96	1	5	0.75	0.8	0.8	0.65	2.5	2	10	0	80	0	0	^b^	200
**R**	154	5	146	1.5	1.5	1	1	0	0–2	10	0	0	0	0	7.4	325

Except in columns labelled pH and osmolality, values are in mM. pH of all media was adjusted with NaOH or HCl without changing final [Na^+^] or [Cl^−^] as tested.

^a^ Numbers in column refer to [H_4_SiO_4_] or [H_4_GeO_4_]. Note that Si is a ubiquitous trace contaminant so that its concentration in the solutions not added with H_4_SiO_4_ was actually ~2–3 μM rather than 0.

^b^ pH was adjusted at either 6.4, 7.4 or 8.4 in 80 mM acetate (to change both extracellular and intracellular pH) or in 80 mM gluconate (to change extracellular pH only [18]).

Abbreviations: Bic, HCO_3_^−^; HEP, HEPES; NMG, *N*-Methyl-D-glucamine; Ace, acetate; Glu, gluconate; Suc, sucrose; Osm, osmolality (mOsM).

#### Studies in *Xenopus laevis* oocytes

To achieve channel expression, oocytes were extracted from *Xenopus laevis* frogs (Nasco—Fort Atkinson, WI), injected with 10–15 ng of cRNA and incubated during 3 days in Barth medium (step 1). In most studies, cells injected with water were used as controls, and in some studies, H_4_SiO_4_ was added during step 1 for Si efflux measurements. After the 3-day incubation, oocytes were subjected to a second incubation of variable duration in distilled water added with ~10 mM sucrose or in different types of modified Barth media added with H_4_SiO_4_ (± H_4_^68^GeO_4_), H_4_GeO_4_ (+ H_4_^68^GeO_4_) or sulfo-NHS, an impermeant biotin that labels cell surface proteins exclusively (step 2). In a few studies, they were incubated a third time in a regular, H_4_SiO_4_-free Barth medium (step 3). For the transport and expression assays *per se*, oocytes were harvested at different times during step 2 or 3 to be assayed for: 1) Si content (by atomic absorption spectrophotometry), 2) ^68^Ge content (by liquid scintillation radioactive counting), 3) cell diameter (by microscopy) or 4) AQP expression by Western blot analyses of cell surface proteins precipitated with streptavidin-coupled DYNAbeads. Technical notes: 1) substrate influx was measured during the linear phase of uptake where transport is largely unidirectional; 2) duration of step 2 was 90 min unless mentioned otherwise; 3) Si content was measured in lysates from 10 oocytes; and 4) Western blot analyses were carried out as previously described [18,19] using cell surface membrane preparations from ~25 oocytes in each lane.

#### Studies in HEK-293 cells

To achieve expression, cells stably-transfected with AQGP were grown to confluence during a 3-day incubation in FBS-added DMEM (step 1). In most studies, cells transfected with the empty vector were used as controls, and in some studies, they were incubated during step 1 in a medium added with H_4_SiO_4_ and siRNAs (Silencer Select Validated siRNA from Ambion) to study the effect of AQP silencing on Si efflux. After the 3-day incubation, HEK-293 cells were subjected to a second 5-min incubation in a physiological medium that was also added in some studies with H_4_GeO_4_ (+H_4_^68^GeO_4_) or H_4_SiO_4_ (step 2). For the transport and expression assays *per se*, HEK-293 cells were harvested at the end of step 2 to be assayed for: 1) Si content (as above), 2) ^68^Ge content (as above) or 3) AQP expression (by conventional qPCR).

### Animal studies

Eighteen C57BL/6 mice were fed a regular diet for three weeks after which they were switched to a Si-depleted or Si-enriched diet (8 or 9 animals per group) for three other weeks (composition of mouse chow was otherwise identical between groups). At the end of the protocol, animals were sacrificed and their *calvarium*, small intestine, kidneys, blood and urine harvested for Si measurements (as described above) or SYBR green-based qPCR studies. Templates used consisted of RNA-derived cDNAs and primers of AQP- or GADPH-specific oligonucleotides (listed in Table 1). Amplification was achieved through 40 PCR cycles set as follows: 95°C for 15 s, 60°C for 30 s and 72°C for 30 s. Templates were used at appropriate dilutions to ensure similar amplification efficiencies between any given AQP and GAPDH templates. Expression studies in normal mammalian tissues were also carried out through EST databank and literature searches as stated in the legend of Table 3. All of the animal studies were approved by the Comité de protection des animaux du Centre hospitalier universitaire de Québec under protocol number 2009127. All efforts were made to minimize suffering.

**Table 3:** Expression of AQPGs and AQP1 in selected mammalian tissues.

	Number of EST transcripts per million				Subtissular and subcellular localization based on previous studies					
AQP	kidney	gut	bone	joint	RTC	SIEC	OB	OC	C&S	refs.
1	584	140	114	1769	+ (PT; BL)	n/a	n/a	n/a	+	[20,21]
3	55	22	0	0	+ (CD; BL)	+ (BL)	+	n/a	+	[21–23]
7	50	20	10	59	+ (PT; AP)	+ (AP)	n/a	n/a	n/a	[24,25]
9	5	13	19	0	n/a	+ (BL)	n/a	+	n/a	[26,27]
10	0	0	0	0	n/a	+ (AP)	n/a	n/a	n/a	[28,29]

Numbers of EST transcripts were obtained through blast searches of human, mouse, rat, dog and bull EST databanks and are expressed per million transcripts based on the following equation: [number of human, mouse, rat, dog and bull AQP transcripts] ÷ [total number of human, mouse, rat, dog and bull AQP transcripts] × 10^6^. Number of AQP-specific and of total transcripts is probably overestimated given that some sequences may belong to the same EST clone. For certain tissues, data were not available in all species and total number of transcripts was low. Subtissular and subcellular localization were obtained through literature searches. Abbreviations: AP, apical; BL, basolateral; CD, collecting duct; C&S, chondrocytes and synoviocytes; n/a, non-available; OB, osteoblasts; OC, osteoclasts; PT, proximal tubule; RTC, renal tubular cells; SIEC, small intestinal epithelial cells.

### Statistics

Data are presented as means ± S.E. from repeated experiments. When used for normalization in a given experiment, data were also from repeated, concurrent determinations. Curve fitting was carried out with a 4-parameter nonlinear logistic equation. Differences between groups of variables were analyzed by ANOVA tests and considered significant at *P* < 0.05.

## Results

Standard transport studies in oocytes are summarized in Fig 1. They show (panel A) that cell surface expression of certain AQPs causes above-background increases in Si transport. Of relevance, Si influx in AQP7-, AQP9- and AQP10-expressing oocytes is quantitatively similar to that in lsi1-expressing oocytes. The transport studies of Fig 1 also show (panel B) that Si uptake in Si-depleted cells and Si loss in Si-loaded cells both saturate with time over short T_1/2_s. For instance, T_1/2(Si uptake)_ for AQP9, AQP10 (curve not shown) and AQP7 were 1, 60 and 300 min, respectively, as would be expected for a substrate that is transported across the oocyte membrane by facilitated diffusion. Otherwise, most of the AQPs tested exhibited water transport activity (panel C) as seen by their ability to produce above-background increases in oocyte volume 20 s after incubation in a ~10-mOsM medium.

**Fig 2:**
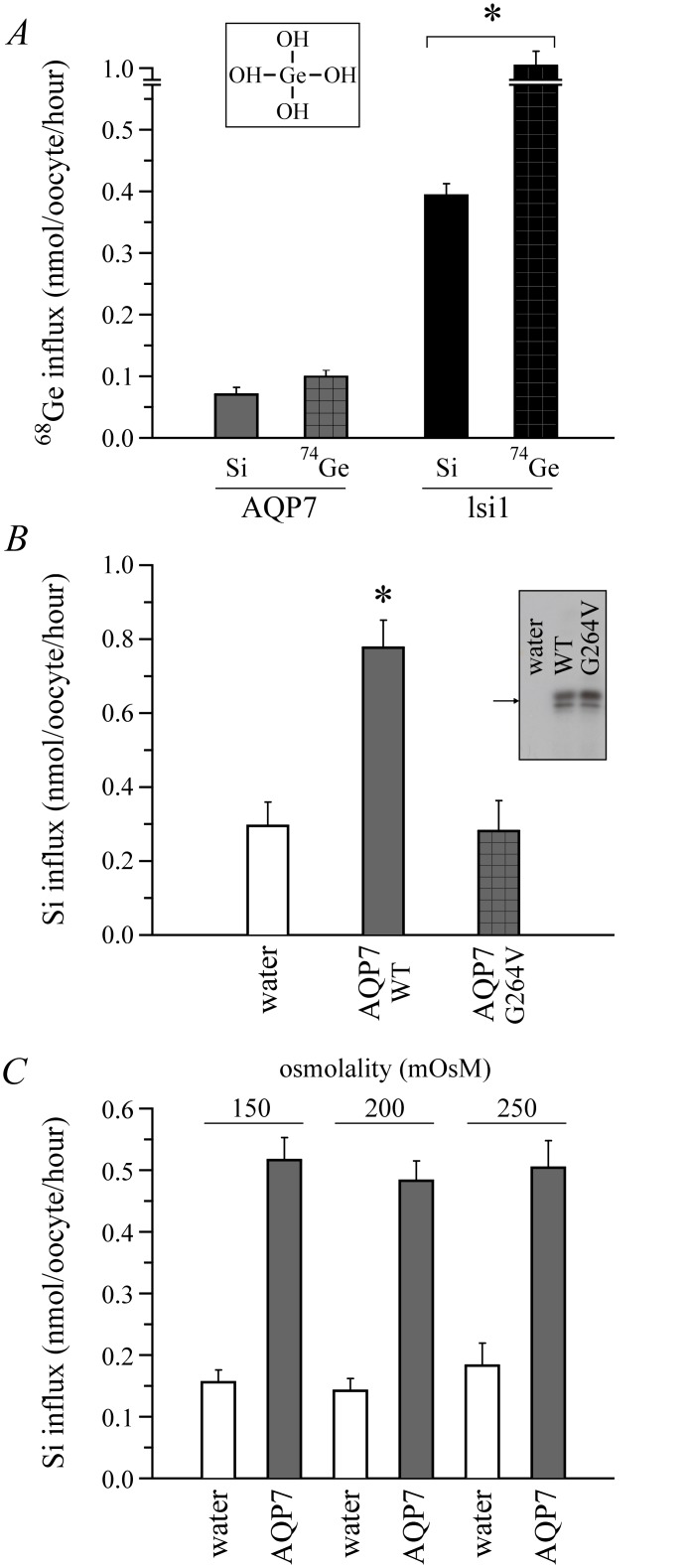
Control Si transport studies in AQP-expressing *Xenopus laevis* oocytes. (A) ^68^Ge influx. Oocytes incubated for 90 min in B1 medium (see Table 2) + 2 mM H_4_GeO_4_ with 1 μCi/mL H_4_^68^GeO_4_ in or + 2 mM H_4_SiO_4_ were assayed for ^68^Ge content. Data correspond to background-subtracted influx values and are presented as means ± S.E. of 12 oocytes among 3 experiments. They were significantly different relative to background, between channels (for a given substrate) and between substrates (for lsi1). The image inserted on the top is to show the chemical structure of H_4_GeO_4_. (B) Si influx in AQP7_G264V_-expressing oocytes. Channels were tagged at the *N*-terminus with the epitope *c-Myc*. Conditions were as described for Fig 1A except that 2 mM sulfo-NHS was added to B1 medium in certain studies. Data are presented as means ± S.E. of 3 measurements among 6–7 experiments using * to indicate that they are significantly different compared to the controls. The image inserted on the right shows that the abundance and migration pattern of cell surface AQP7_G264V_ by Western blot analysis are similar to those of wild type AQP7. (C) Dependence of Si influx on external osmolality (or on net water transport). Oocytes incubated for 90 min in a modified B1 medium (B2a, B2b or B2c) were assayed for Si content. Data are presented as means ± S.E. of 3 measurements among 6 experiments. Influx values among conditions are not significantly different. Under isotonic conditions, cell volumes after 20 s were also similar (885 ± 72 vs. 791 ± 97 nL). Abbreviation: WT, wild type.

Additional transport studies in oocytes (using AQP7 as a model) confirmed that Si transport was directly mediated by the AQGPs. They are presented in Fig 2. For instance, panel A shows that AQP7 promotes above-background influx of radioactive ^68^Ge, a Si surrogate [16,30], in both ^74^Ge- and Si-supplemented media. Along the same line, panel B shows that AQP7_G264V_, a water channel that is known to be inactive [31], does not lead to above-background Si accumulation but is able to reach the cell surface. Lastly, panel C shows that Si influx in AQP7-expressing cells is not altered through maneuvers that affect water transport such as a change in cell volume. Taken together, the data of Fig 2 indicate that Si accumulation in oocytes is unlikely to be contaminated by a component of non-transported, channel-bound substrate or a component of solvent drag-induced substrate transfer. As indicated in Fig legend 2C, in addition, AQP7-expressing oocytes did not undergo net volume changes under isotonic conditions, arguing further against a solvent drag effect.

**Fig 3:**
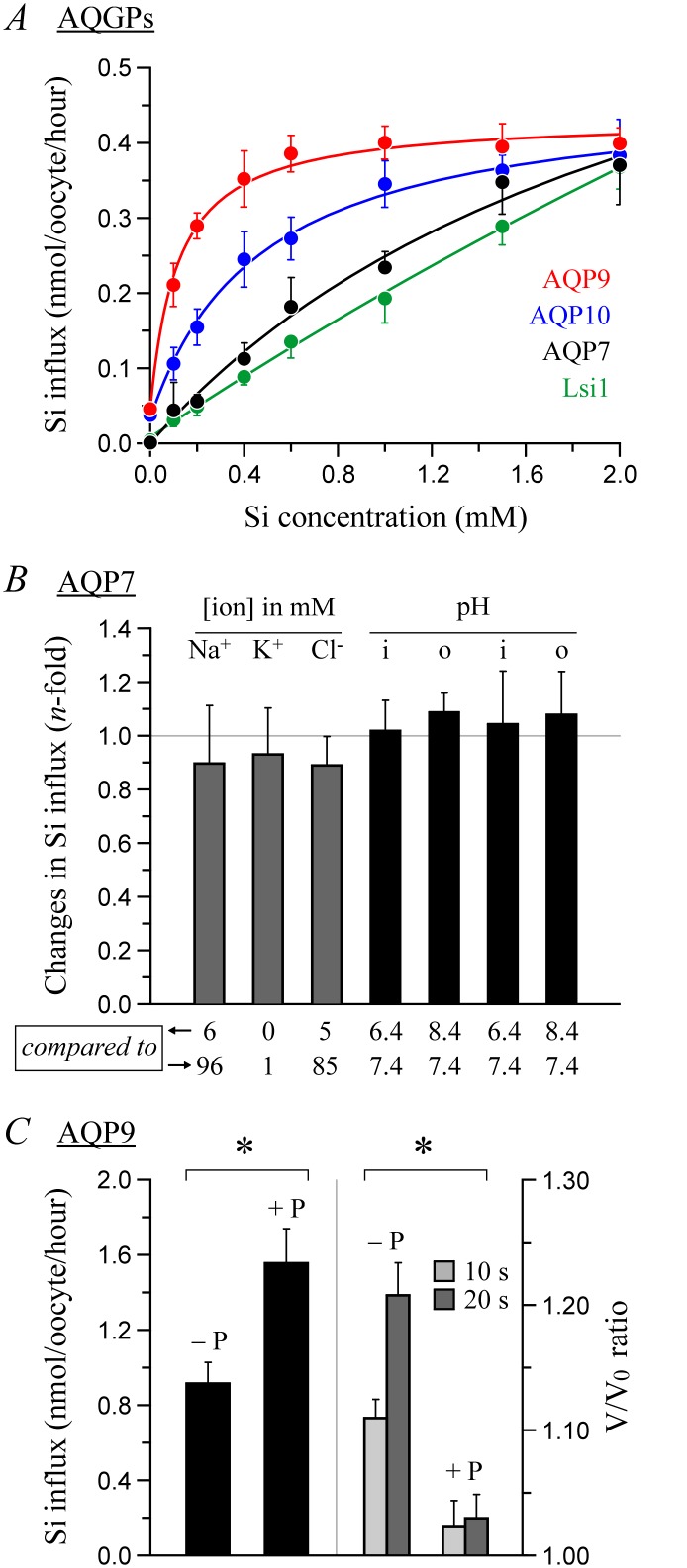
Characteristics of Si transport by AQP-expressing *Xenopus laevis* oocytes. (A) Dependence of Si influx on [H_4_SiO_4_]. Oocytes incubated for 30 min (AQP9), 60 min (AQP10) or 90 min (AQP7 and lsi1) in B1 medium (see Table 2) + 0 to 2 mM H_4_SiO_4_ were assayed for Si content. Data are expressed as background-subtracted influx values and are presented as means ± S.E. of 3 measurements among 3–5 experiments. (B) Dependence of Si influx on intracellular pH and on extracellular pH, [Na^+^], [K^+^], and [Cl^−^]. Oocytes incubated for 90 min in a modified B1 medium (called medium B3a, B3b, B3c, B3d or B3e) + 2 mM H_4_SiO_4_ were assayed for Si content. Data are expressed as *n*-fold differences and presented as means ± S.E. of 3 measurements among 6 experiments. None of the data are significantly different from 1. (C) Effect of phloretin on Si influx and water transport. Left scale: Oocytes incubated for 30 min in B2a medium + 2 mM H_4_SiO_4_ ± 0.1 mM phloretin were assayed for Si content. Data are expressed as background-subtracted influx values and presented as means ± S.E. of 3 measurements among 6 experiments. Right scale: Oocytes were assayed for cell volume during a 20-s incubation in ~10 mM sucrose ± 0.1 mM phloretin. Data are expressed as *n*-fold increases in cell volumes (V) relative to initial cell volumes (V_0_) and presented as means ± S.E. of 5 oocytes among 4 experiments. * indicates that the values are significantly different between − P and + P. Abbreviations: i, intracellular; o, extracellular; + P, phloretin.

A more detailed characterization of Si transport by certain AQPs is presented in Fig 3. As can be observed in panel A, Si influx by AQP7, AQP9 and AQP10 increases as a function of external [Si], but exhibits nonlinear kinetics so as to yield half-rate concentrations of 2300, 117 and 470 μM, respectively. Contrastingly, Si influx by lsi1 is characterized by nearly linear kinetics as described previously [16]. Otherwise, Si transport by AQP7 is seen to be unaffected by changes in intracellular pH or in extracellular pH, [Rb^+^], [Na^+^] or [Cl^−^] (panel B) and Si transport by AQP9 is seen to be stimulated by the water transport inhibitor phloretin [14] under hypoosmolar conditions (panel C). These characteristics suggest further that Si is directly transported by the expressed channels. In conjunction with the data of Fig 2C, they also imply that Si could interfere with the passage of other substrates through the pore. In this regard, one would have expected higher extracellular-to-intracellular [Si] gradients (as induced through cell swelling) to increase Si transport, and lower gradients (as induced through cell shrinkage), to decrease it.

**Fig 4:**
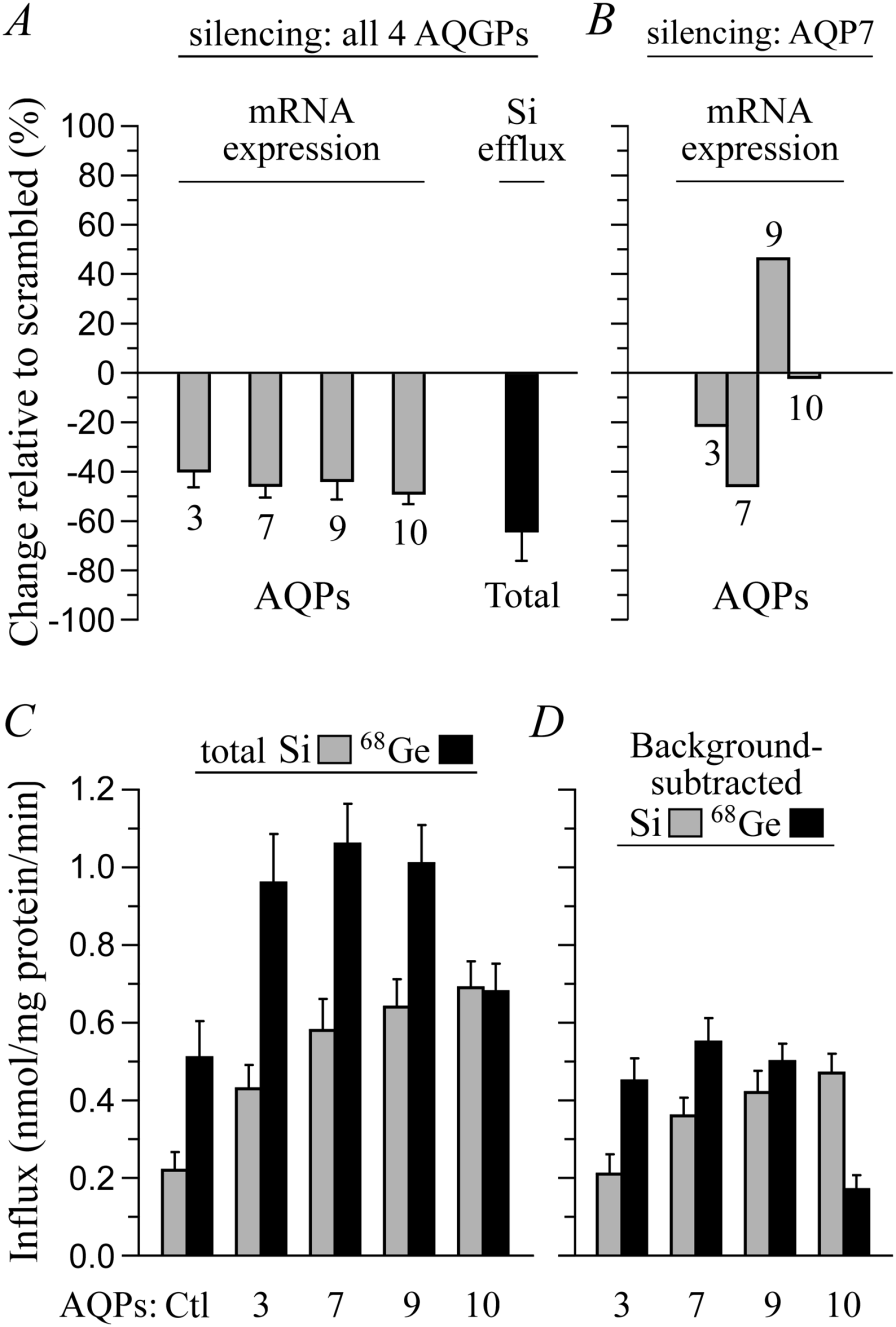
AQP expression and Si transport in HEK-293 cells. (A) Effect of anti-AQP3, AQP7, AQP9 and AQP10 siRNAs on AQPG expression and Si efflux. HEK-293 cells incubated for ~48 h in R medium (see Table 2) + 2 mM H_4_SiO_4_ ± 5 different siRNAs and for 5 additional min in R medium alone were assayed for AQP expression by qPCR and for Si content. Data are expressed as % changes between conditions “scrambled siRNAs” and “anti-AQP siRNAs” and are presented as means ± S.E. of 2–8 measurements among 3 experiments. All of the changes are significantly different from 0. Note that threshold cycles in the absence of siRNAs were of ~30 for all the AQGPs. (B) Effect of anti-AQP7 siRNAs on AQPG expression. Protocols used and data expression are as described for panel A. Except for AQP10, all of the changes are significantly different from 0. (C) Total Si and ^68^Ge influx. AQGP-transfected HEK-293 cells incubated for 5 min in R medium + 2 mM H_4_GeO_4_ with 1 μCi/mL H_4_^68^GeO_4_ or in R medium + 2 mM H_4_SiO_4_ were assayed for both ^68^Ge or Si content, respectively. Data are expressed as mean influx values ± S.E. of 4–8 measurements among 3–5 experiments. Compared to the controls, all of the values are significantly different. (D) Background-subtracted ^68^Ge and Si influx. Influx values measured in pCDNA-transfected HEK-293 cells were subtracted from the influx values measured in AQGP-transfected HEK-293 cells. All values are significantly different from 0.

Transport studies to determine whether both native and heterologous AQGPs could play the role of Si transporters in mammalian cells are summarized in Fig 4. HEK-293 cells were chosen for these experiments given that they express all four AQGPs and were thus expected to exhibit sizeable endogenous Si transport activity. It is seen that reduced expression of all four channels (through multiple RNA interference) leads to a substantial reduction in Si efflux (panel A), and that heterologous expression of either channel leads to substantial increases in Si as well as ^68^Ge influx (panels C and D). In these experiments, interestingly, ^68^Ge and Si influx tended to differ for any given isoform, perhaps because of variant affinities for the substrates, and inhibition of one isoform was often found to increase that of the other isoforms (see example in panel B with anti-AQP7 siRNAs), perhaps as a regulatory response to subtle changes in intracellular osmolality.

Previous localization studies have shown that certain AQPs were expressed in renal and small intestinal epithelia, both of which should play a key role in Si balance regulation, and that they were also expressed in bone and joint, both of which appear to require Si for optimal development or function [10–14,17]. EST databank and Medline searches were thus carried out to determine whether Si-transporting AQPs could be involved in such processes based on transcript abundance and a more exhaustive review of literature. Results, which are summarized in Table 3, corroborate this possibility given that the surface of renal epithelial cells, small intestinal epithelial cells and bone turnover cells each express two AQGPs or more at relatively high levels [20–29].

**Fig 5:**
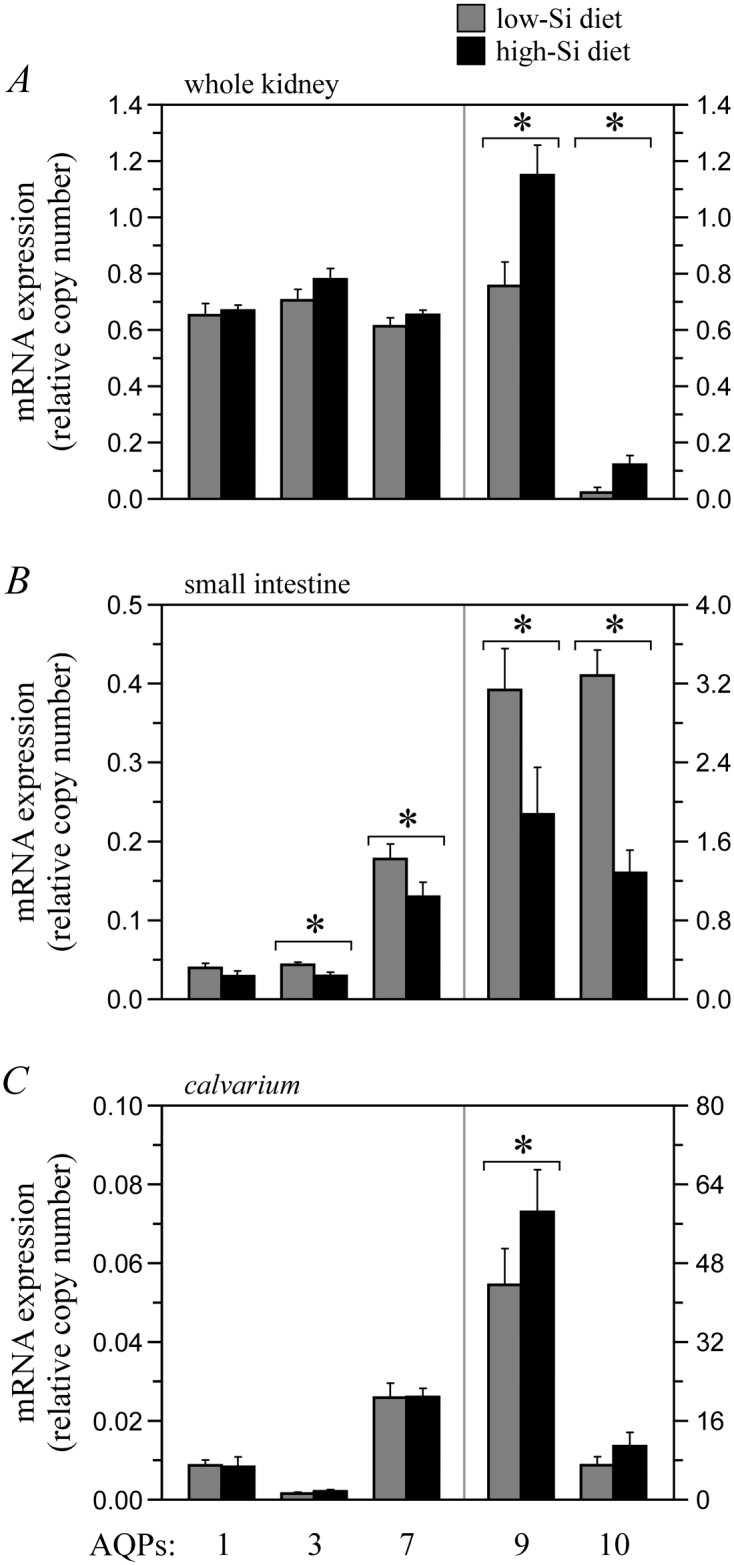
Effect of high- and low-Si diets on AQP transcription in selected mouse tissues. Animals were subjected to a Si-rich diet (4% elementary Si; *n* = 9) or a Si-poor diet (0.4% elementary Si; *n* = 8 or 9) for 3 weeks and sacrificed afterwards to carry out qPCR studies using total RNA from kidney (panel A), small intestine (panel B) or *calvarium* (panel C) as templates. Oligonucleotides used are shown in Table 1. Data are expressed as AQP-specific DNA copy numbers normalized to GADPH-specific DNA copy numbers and presented as means ± S.E. of 8–9 mice between two experiments using * to indicate that they are significantly different compared to each other. Of notice, relative AQP1 expression levels were lower than expected based on the data of Table 3 so that conditions used to amplify this isoform might have been suboptimal. Under the Si-rich diet, in addition, blood and urine [Si] were over 10-fold higher compared to the Si-poor diet.

In a final series of experiments conducted to determine whether AQP-mediated Si transport is of potential physiological relevance, we compared the effect of administering Si-poor and Si-rich diets during 3 weeks on channel transcription in mouse kidney, small intestine and *calvarium*. As illustrated through Fig 5, expression of several AQPs in these tissues was found to be not only abundant, but diet-dependent as well. Under the Si-rich diet, indeed, mRNA levels for AQP9 and AQP10 are seen to be higher in kidney and *calvarium* (panel A and C) and mRNA levels for all AQGPs are seen to be lower in small intestine (panel B). These observations are consistent with the idea that some of the AQP family members play a role in Si balance regulation.

## Discussion

A number of the AQPs studied in this work have been found to play a novel and potentially pivotal function in mammals, i.e., that of acting as ubiquitously distributed, regulatable Si transport systems at the cell surface. For instance, Si influx in AQP9- and AQP10-expressing oocytes rose as a function of extracellular [Si] according to saturable kinetics and reached half-rates over potentially relevant [Si] ranges [3–5]. Along the same line, the transcriptional activity of certain AQPs in selected mouse tissues was not only sensitive to changes in dietary Si, but it also varied according to expectation in small intestine (Fig 5), consistent with the existence of Si responsive elements [13–15]. Studies are underway to study AQP expression at the protein level, but none of the commercially available antibodies used thus far have been sufficiently sensitive or specific to allow for precise quantitative determinations.

As stated earlier, the water channels found to display Si transport activity were all members of the AQGP subfamily. Whether conventional AQP or S-AQP family members could also act as Si transporters is currently unknown given that this possibility has been either not tested (AQP0, AQP4, AQP5, AQP6 and AQP12) or could have been tested under suboptimal conditions (AQP1, AQP2, AQP8 and AQP11). In this regard, interestingly, AQGP-mediated Si movement was found to increase in the presence of phloretin, a flavonoid that affects the activity of many transport systems.

According to our kinetic data, subsaturating [Si] for the AQGPs tested were all higher than 10 to 50 μM, i.e., than the normal [Si] of adult human serum [3–5]. Yet, the physiological relevance of Si transport by the AQGPs cannot be excluded for this reason. First, subsaturating [Si] for AQP9 was still close to 50 μM. Second, [Si] in human serum is much higher during early infancy, pregnancy, high-Si diets and stage-V renal failure, and [Si] in urine and connective tissues can reach the submillimolar range [3–5]. Lastly, a number of transport systems are physiologically relevant even though substrate influx is known to be poorly saturable [32,33].

For lsi1, Ma *et al*. [16] found Si transport as a function of [Si] to be linear between 0 to 2 mM rather than at least partly or completely saturable as reported in this study for the human AQGPs. Although plant and human channels were characterized in the same expression system, Ma *et al*. used ^68^Ge as a tracer instead of measuring Si directly and presented no data to determine whether substrate uptake at 30 min was in the linear phase of transport. It should be mentioned, in addition, that the kinetic characteristics of substrate movement by a transport system can vary greatly among orthologs and among transportable substitutes [18,19].

In this work, control data obtained to ensure that Si content in oocytes did not change through solvent drag were conclusive. Those obtained to exclude a substantial AQP-bound component were also convincing. For instance, we found that Si influx in oocytes expressing plant lsi1, a channel that promotes Si transfer from soil to phloem [16], was quantitatively similar compared to Si influx in oocytes expressing AQP7, AQP9 and AQP10. Along the same line, we found that an inactive AQP7 mutant that can still reach the oocyte surface was unable to promote Si accumulation. Based on such data, on the effect of anti-AQGP siRNAs in HEK-293 cells, and on the observation that another metalloid (arsenite) behaves as a transported substrate for the AQGPs [17], it also appears unlikely that channel expression in oocytes could have led to the activation of endogenous metalloid pathways through cell swelling or other indirect functional changes.

The detailed characterizing of Si transport by the AQGPs in oocytes also pointed towards a scenario where Si and water could influence the movement of each other through the pore. For instance, Si content failed to decrease in AQP7-expressing oocytes at higher extracellular osmolality (Fig 2C) and in phloretin-treated AQP9-expressing oocytes at lower osmolality (Fig 3C), i.e., under conditions where water uptake is decreased. Given that the AQPs can transport a wide variety of substrates besides water, and that these substrates are covered by hydration shells in solution—this is the case for H_4_SiO_4_ in particular—it is tempting to postulate that water transport by some of these channels occurs to some extent in the form of hydrated substrates from which shell layers can be shed or added [17,34–37]. It is also tempting to postulate that phloretin acts upon certain AQPs by altering the conformation of the pore through allosteric effects.

Over the years, failure to identify transport systems for Si in mammals has probably contributed to undermine an important field of investigation. Our data could thus represent an important first step towards unveiling the elusive and debated role of Si in a number of physiological processes. For example, they could find direct relevance in osteoporosis based on the observations that Si interferes with aluminium deposition at the bone mineralization front [7,9], that bone turnover cells express certain AQGPs and that bone metabolism is impaired in AQP1^−/−^ [38] and AQP9^−/−^ mice [39]. They could also find relevance in uraemia-associated vascular calcifications or rigidity based on the observation that the kidney is a major elimination organ for Si [13]. In these disorders, intriguingly, mineralization defects have been attributed mostly to Ca^2+^ deposition defects when in fact, other minerals such as Si are known to interact with collagen-based matrices and contribute to tissue rigidity [40].

In conclusion, we have identified Si transport systems in mammals for the first time, uncovered a novel role for the AQGPs and proposed a transport model for substrate movement by water channels. Even today, hence, the discovery of AQPs by Agre *et al*. appears to remain a key premise from which additional and long-standing biological questions can be answered. In this sense, it might also prove central in determining whether Si, which has become an important supplement in the highly lucrative market of natural products, is endowed with many of its claimed therapeutic virtues.
